# Belief in omens and superstitions among patients with chronic neurological disorders

**DOI:** 10.3389/fpubh.2024.1331254

**Published:** 2024-03-07

**Authors:** Rūta Mameniškienė, Rasa Kizlaitienė, Rūta Kaladytė Lokominienė, Kristijonas Puteikis

**Affiliations:** ^1^Centre for Neurology, Faculty of Medicine, Vilnius University, Vilnius, Lithuania; ^2^Faculty of Medicine, Vilnius University, Vilnius, Lithuania

**Keywords:** epilepsy, multiple sclerosis, neurology, Parkinson’s disease, religious belief, spirituality

## Abstract

**Introduction:**

Chronic neurological disorders may affect various cognitive processes, including religiosity or superstitious belief. We investigated whether superstitious beliefs are equally prevalent in patients with Parkinson’s disease (PD), people with epilepsy (PWE), patients with multiple sclerosis (MS) and healthy controls (HCs).

**Methods:**

From late 2014 to early 2023 we conducted a cross-sectional in-person anonymous paper-based survey at the tertiary clinic of Vilnius University Hospital Santaros Klinikos among outpatients and HCs by asking them to ascribe meaning or report belief for 27 culturally adapted statements (9 omens and 18 superstitions). The sum of items that a respondent believes in was labeled the superstition index (SI). The SI was compared between groups by means of the Kruskal-Wallis (H) test and negative binomial regression modeling. A two-step cluster analysis was performed to discern different subgroups based on answers to the items of the SI.

**Results:**

There were 553 respondents who completed the questionnaire (183 PWE, 124 patients with PD, 133 with MS and 113 HCs). Complete SI scores were collected for 479 (86.6%) participants and they were lower in patients with PD (*n* = 96, Md = 1, IQR = 0–5.75) in comparison to those with epilepsy (*n* = 155, Md = 6, IQR = 1–14), MS (*n* = 120, Md = 4, IQR = 0–12) or HCs (*n* = 108, Md = 4.5, IQR = 1–10), H (3) = 26.780, *p* < 0.001. In a negative binomial regression model (*n* = 394, likelihood ratio *χ*^2^ = 35.178, p < 0.001), adjusted for sex, place of residence, income and education, female sex was the only characteristic associated with the SI (*β* = 0.423, OR = 1.526, 95% CI = 1.148 to 2.028). Both female sex (*β* = 0.422, OR = 1.525, 95% CI = 1.148 to 2.026) and Parkinson’s disease (*β* = −0.428, OR = 0.652, 95% CI = 0.432 to 0.984) were significant predictors of the SI when age was removed from the model. Two-step cluster analysis resulted in individuals with PD being grouped into “extreme non-believer,” “non-believer” and “believer” rather than “non-believer” and “believer” clusters characteristic for PWE, patients with MS and HCs.

**Conclusion:**

Our study suggests that individuals with PD believe in less superstitions than patients with MS, PWE or HCs. The results of this investigation should be independently confirmed after adjusting for PD-specific variables.

## 1 Introduction

Epilepsy, Parkinson’s disease and multiple sclerosis are chronic neurological disorders with age-standardized global prevalence of 327, 94 and 30 per 100,000 individuals, respectively, according to the Global Burden of Disease Study 2016 (1). All three disorders lead to greater disability and premature death both in European populations and worldwide (1–3). Beyond medical implications, such chronic diseases are also associated with greater healthcare and personal costs, difficulties to lead a socially active life as well as an increased risk for mental health disorders, such as depression and anxiety (4, 5). As many other cognitive processes, the presence of superstitious belief may be supposed to be influenced by brain lesions, neuronal network disruptions or neurodegeneration resulting from such chronic neurological disorders. Differences of belief in superstition may also occur because of the dissimilar demographic, clinical, psychological and societal aspects associated with all three conditions. Despite the possible variance in superstitious belief across the selected neurological diseases, research of belief among patients suffering from them has been focused mostly on religiosity or spirituality. For instance, it has been posited that individuals with Parkinson’s disease (PD) may be less religious and spiritually minded than healthy ones, probably because of disruption of dopaminergic pathways (6, 7). People with epilepsy (PWE), on the other hand, have been investigated for increased religiosity and spirituality that can be associated with spiritual experiences during seizures and/or structural changes of regions like the temporal lobe (8, 9). PWE have also been thought to develop “hyperreligiosity” and “hypergraphia” as behavior traits of an “epileptic personality” – such a view is now considered to be discriminatory and reductionistic (10, 11). However, the extent of superstitious beliefs in epilepsy, PD and MS has received little to no attention in the literature over the years. We believe it is an interesting research question to address, given the debatable impact of neurological disease on other aspects of patient belief systems that have been investigated previously.

Paranormal, superstitious, magical and supernatural beliefs are phenomena that can be defined by misattribution of properties of one ontological category to another (e.g., the attribution of intentionality to random events leads to the belief in fate) (12). According to Lindeman and Svedholm who proposed this definition, there is no substantial difference among the terms paranormal, superstitious, magical and supernatural. However, “superstition” has been more used in Western research of the formation and maintenance of irrational behaviors and tends to approach belief objects like lucky numbers, luck-related rituals, charms, omens, fate and illusory rules (12). The definition of superstition may also be narrowed down to encompass category mistakes that include presumption of causal relationship (e.g., between a sign or action and luck or future (mis)fortune) (13).

While the content of superstitious reasoning can emerge from both personal (e.g., a team supporter’s rituals before an important sports match) and cultural (e.g., avoidance of the number “13”) settings, the tendency to form and sustain superstitions is thought to be rooted in human psychology. Risen suggested a refined dual process theory that explains how superstitions can be so widespread and persist even among well-educated and otherwise rational individuals (13). This theory incorporates the “quick and effortless” System 1 (its key components leading to superstitious thinking are heuristics and attribute substitution, causal intuitions and confirmation bias) that can be overridden by the “slower and effortful” System 2 (its key components are the ability and motivation to be rational as well as contextual cues) when an irrational intuition is activated (13–15). Within this framework, the irrational intuition may remain uncorrected even if System 2 becomes engaged and the individual knows that his/her reasoning is unfounded. In this case, individuals may choose to acquiesce to their intuition (e.g., because the resulting decision is more compelling or has no significant cost) (13). This theory helps to explain the great prevalence of superstitious beliefs in the general population and regard it as a normal occurrence that is generally not indicative of some mental or neurological disorder (16, 17).

Religion and spirituality manifest as personal and/or organizational acceptance of a multifaceted belief system that usually has deep historical and cultural roots and is a different and more complex construct than superstitions (18). The latter, on the other hand, may be perceived as simpler erroneous by-products of the brain’s processing of causality and contingency (19). The understanding of superstitious beliefs as cognitive and/or behavioral phenomena possibly resulting from various cognitive biases is expected to make their research less reductionistic and less confounded by cultural or historical factors than in the case of religion and spirituality. Moreover, the investigation of superstitions in individuals with neurological disorders may help to better understand the structural and/or functional substrate of such irrational beliefs (20).

The current study was designed to explore the prevalence of superstitions among patients with three chronic neurological disorders: PD, multiple sclerosis (MS) and epilepsy. Based on previous findings across literature that are described above, we aimed to test the hypotheses that patients with PD are less superstitious than patients with MS, PWE, or healthy controls, HCs (H_1_), and that PWE are more superstitious than any other study group (H_2_).

## 2 Methods

### 2.1 Study setting

A cross-sectional study was conducted from late 2014 to early 2023 at a tertiary outpatient neurology clinic at Vilnius University Hospital Santaros Klinikos and included three groups of patients having either epilepsy, MS or PD as well as a group of HCs (composed of colleagues and healthy patients’ relatives). The long enrolment period was caused by rather low patient involvement during time-constrained routine medical visits as well as disruptions of the COVID-19 pandemic in years 2020–2021. Participants were offered to complete an anonymous paper-based survey about their beliefs in omens and superstitions as they arrived for routine medical visits in person. Given the exploratory nature of the study, the three disorders were selected *ad hoc* based on the notion that they all have impact on cognitive functioning (and, thus, may influence superstitious cognitive processing) and the capacity of outpatient visits was deemed sufficient to collect the required sample size across respective patient groups. The inclusion criteria for patients were: (1) to have a previously established diagnosis of one of the aforementioned disorders, (2) to undergo treatment in an attempt to mitigate the disorder (i.e., newly diagnosed cases were excluded), (3) to be ≥18 years old, (4) to understand the questionnaire in Lithuanian and (5) to have no comorbid psychiatric disorder. Patients not speaking Lithuanian, otherwise unable or unwilling to participate after being explained the study design and aims were excluded. The survey was designed to be anonymous as no person-identifying data was collected and individual forms were not retraceable to individual respondents.

### 2.2 The questionnaire

The questionnaire consisted of two parts:

The first part addressed demographic variables (sex, age, place of birth, place of residence, relationship status, number of children, education, employment) and socioeconomic status (main source of income, average monthly income). In this part, respondents also reported general religious or spiritual beliefs:

- Whether they believe in God or “a higher power”
- How often they attend church
- How often they pray at home
- Whether they believe in horoscopes
- Whether they have ever visited a fortune-teller
- Whether they believe some days are luckier than other
- Whether they believe that there are signs predicting future misfortune

The second part of the survey form consisted of a list of 9 signs (omens) and 18 superstitious statements (Supplementary Appendix). Respondents were asked to select whether the signs are “good,” “bad” or “have no meaning” and whether they believe in the listed superstitions (“Yes” or “No”). As no literature about the frequency of various superstitions in the general population of Lithuania was identified, the great variety of omens, superstitions and their different interpretations prompted us to select those reoccurring in Lithuanian news articles and websites and judged by the authors to be understandable, relatable, socially appropriate and encountered at least once before the current study. During such an assessment for face validity, omens and superstitions including religious or spiritual themes were avoided to prevent their overlap with the conceptual framework of superstitiousness.

### 2.3 Statistical analysis

IBM SPSS v26 and MS Excel v2206 were used for statistical analysis. Chi-square or Fisher’s exact (Monte Carlo method) tests were used to compare the distribution of categorical values between groups. Data from the second part of the questionnaire was quantified by calculating the sum of omens respondents ascribed either positive or negative meaning and superstitions they reportedly believe in. This sum was labeled the superstitiousness index (SI) and treated as a count variable with Poisson distribution. The SI was compared between participant groups and among their subgroups based on demographic and socioeconomic variables by using Mann Whitney U and Kruskal-Wallis (H) tests. The association between the SI and ordinal or continuous variables was determined by calculating Spearman’s correlation coefficients. Variables found to be associated with the SI in a statistically significant way were then entered in a Poisson loglinear regression model as independent variables. The latter was switched to a negative binomial model in case of overdispersion (Pearson χ^2^/degrees of freedom(df) > 1.2) (21). The required sample size was approximated to be 280 based on a one-way analysis of variance for four participant groups, medium effect size *f* = 0.25, *α* = 0.05 and *β* = 0.95 (G*Power 3.1.9.7).

Binary items comprising the SI were also explored by performing a two-step cluster analysis that provided information about respondent grouping based on either their belief or disbelief in the listed omens or superstitions. This analysis was first conducted with the whole study sample and then with individual patient or control groups. The quality of the cluster was evaluated by the silhouette measure of cohesion and separation (≤0.25 perceived as poor, 0.26–0.50 as fair and > 0.50 as good) (22).

### 2.4 Ethics statement

Anonymized surveys are not considered to be biomedical studies by Lithuanian law (Article No. 3 “Objects of biomedical research” of the Law on the Ethics of Biomedical Research of the Republic of Lithuania No. VIII-1679) and its interpretation by the Lithuanian Bioethics Committee (Čekanauskaitė A, Peičius E, Urbonas G, Lukaševičienė V. Public Health. 2021;2(93):72), therefore the study was exempt from bioethical review. The study and all methods were carried out in accordance with relevant guidelines and regulations, including the World Medical Association Declaration of Helsinki. Written informed consent was obtained from all participants with the survey form. All participants remained anonymous throughout the study.

## 3 Results

The study sample consisted of 183 individuals with epilepsy, 124 with PD, 133 with MS and 113 HCs. The characteristics of the study participants are presented in Table 1 while their religious or spiritual beliefs as well as beliefs in omens or superstitions are shown in Tables 2, 3.

**Table 1:** Characteristics of the study sample.

**Characteristic**	**Epilepsy (*n* = 183)**	**PD (*n* = 124)**	**MS (*n* = 133)**	**HC (*n* = 113)**	**Test and *p* value**
**Age, years (mean, SD)**	36.8 (15.3)	63.9 (11.2)	42.3 (12.4)	33.6 (15.7)	Z = 186.3, p < 0.001**
**Sex (*n*, %)**					16.247, *p* = 0.001*
Male	75 (41)	57 (46)	33 (24.8)	47 (41.6)	
Female	101 (55.2)	59 (47.6)	97 (72.9)	66 (58.4)	
Missing	7 (3.8)	8 (6.5)	3 (2.3)	0 (0)	
**Place of birth (*n*, %)**					14.869, *p* = 0.021*
City	112 (61.2)	52 (41.9)	72 (54.1)	69 (61.1)	
Town	26 (14.2)	27 (21.8)	24 (18)	23 (20.4)	
Village	38 (20.8)	40 (32.3)	32 (24.1)	20 (17.7)	
Missing	7 (3.8)	5 (4)	5 (3.8)	1 (0.9)	
**Place of residence (*n*, %)**					10.505, *p* = 0.105
City	126 (68.9)	95 (76.6)	100 (75.2)	95 (84.1)	
Town	22 (12)	15 (12.1)	11 (8.3)	7 (6.2)	
Village	28 (15.3)	9 (7.3)	17 (12.8)	10 (8.8)	
Missing	7 (3.8)	5 (4)	5 (3.8)	1 (0.9)	
**Relationship status** ^**a**^ **(*n*, %)**					105.821, *p* < 0.001**
Not married	67 (36.6)	8 (6.5)	32 (24.1)	53 (46.9)	
Married	71 (38.8)	82 (66.1)	75 (56.4)	37 (32.7)	
Divorced	18 (9.8)	18 (14.5)	6 (4.5)	6 (5.3)	
Widow	7 (3.8)	15 (12.1)	6 (4.5)	3 (2.7)	
Partnered (without marriage)	18 (9.8)	0 (0)	12 (9)	13 (11.5)	
Missing	2 (1.1)	1 (0.8)	2 (1.5)	1 (0.9)	
**Children** ^**a**^ **(*n*, %)**					66.710, *p* < 0.001**
None	89 (48.6)	24 (19.4)	43 (32.3)	69 (61.1)	
1	37 (20.2)	24 (19.4)	38 (28.6)	14 (12.4)	
2–3	50 (27.3)	66 (53.2)	43 (32.3)	23 (20.4)	
4–6	2 (1.1)	3 (2.4)	4 (3.0)	4 (3.5)	
Missing	5 (2.7)	7 (5.6)	5 (3.8)	3 (2.7)	
**Education**^**a**^ **(*n*, %)**					123.305, *p* < 0.001**
Primary	3 (1.6)	1 (0.8)	0 (0)	1 (0.9)	
Lower secondary	17 (9.3)	5 (4.0)	3 (2.3)	2 (1.8)	
Upper secondary	45 (24.6)	9 (7.3)	8 (6.0)	55 (48.7)	
Vocational education	26 (14.2)	12 (9.7)	20 (15.0)	5 (4.4)	
Post-secondary non-tertiary	23 (12.6)	30 (24.2)	20 (15.0)	7 (6.2)	
Tertiary (non-university)	26 (14.2)	10 (8.1)	22 (16.5)	7 (6.2)	
Tertiary (university)	42 (23)	56 (45.2)	60 (45.1)	34 (30.1)	
Missing	1 (0.5)	1 (0.8)	0 (0)	2 (1.8)	
**Employment (*n*, %)**					53.155, *p* < 0.001**
Employed full-time	72 (39.3)	28 (22.6)	72 (54.1)	46 (40.7)	
Employed part-time	14 (7.7)	15 (12.1)	14 (10.5)	15 (13.3)	
Unemployed (in search of employment)	36 (19.7)	6 (4.8)	13 (9.8)	11 (9.7)	
Unemployed (not seeking employment)	56 (30.6)	68 (54.8)	33 (24.8)	41 (36.3)	
Missing	5 (2.7)	7 (5.6)	1 (0.8)	0 (0)	
**Main source of income (*n*, %)**					151.628, *p* < 0.001**
Salary	81 (44.3)	43 (34.7)	78 (58.6)	54 (47.8)	
Pension	38 (20.8)	70 (56.5)	32 (24.1)	8 (7.1)	
Social allowance	33 (18.0)	7 (5.6)	11 (8.3)	4 (3.5)	
Other	29 (15.8)	3 (2.4)	11 (8.3)	47 (41.6)	
Missing	2 (1.1)	1 (0.8)	1 (0.8)	0 (0)	
**Average net monthly income (euros)** ^**a**^ **(*n*, %)**					23.063, p = 0.021*
<250	70 (38.3)	25 (20.2)	28 (21.1)	31 (27.4)	
250–500	42 (23)	34 (27.4)	30 (22.6)	38 (33.6)	
500–1,000	35 (19.1)	41 (33.1)	20 (15.0)	26 (23.0)	
1,000–2000	14 (7.7)	16 (12.9)	17 (12.8)	13 (11.5)	
>2000	6 (3.3)	3 (2.4)	3 (2.3)	3 (2.7)	
Missing	16 (8.7)	5 (4.0)	35 (26.3)	2 (1.8)	

HCs, healthy controls; MS, multiple sclerosis; PD, Parkinson’s disease; ^a^results of Monte Carlo test are presented; *p < 0.05; **p < 0.001.

**Table 2:** General beliefs of the study participants.

**Question**	**Epilepsy (*n* = 183)**	**PD (*n* = 124)**	**MS (*n* = 133)**	**HC (*n* = 113)**	**Test and *p* value**
**Do you believe in God?**					8.483, *p* = 0.205
Yes, I believe in God	104 (56.8)	79 (63.7)	67 (50.4)	56 (49.6)	
Yes, I believe there is a higher power	41 (22.4)	29 (23.4)	36 (27.1)	38 (33.6)	
No	34 (18.6)	15 (12.1)	22 (16.5)	17 (15)	
Missing	4 (2.2)	1 (0.8)	8 (6)	2 (1.8)	
**How often do you go to church?** ^**a**^					16.708 *p* = 0.053
Once or several times per week	10 (5.5)	5 (4)	6 (4.5)	6 (5.3)	
Once or several times per month	29 (15.8)	33 (26.6)	17 (12.8)	20 (17.7)	
Once or several times per year	88 (48.1)	58 (46.8)	74 (55.6)	66 (58.4)	
I do not go to church	54 (29.5)	25 (20.2)	33 (24.8)	18 (15.9)	
Missing	2 (1.1)	3 (2.4)	3 (2.3)	3 (2.7)	
**Do you pray at home?**					17.844, *p* = 0.037*
Every day	16 (8.7)	16 (12.9)	11 (8.3)	14 (12.4)	
Often	20 (10.9)	13 (10.5)	12 (9)	12 (10.6)	
Sometimes	50 (27.3)	53 (42.7)	47 (35.3)	27 (23.9)	
No	97 (53)	42 (33.9)	62 (46.6)	60 (53.1)	
Missing	0 (0)	0 (0)	1 (0.8)	0 (0)	
**Do you believe in horoscopes?**					20.046, p < 0.001**
Yes	56 (30.6)	11 (8.9)	30 (22.6)	25 (22.1)	
No	120 (65.6)	106 (85.5)	100 (75.2)	86 (76.1)	
Missing	7 (3.8)	7 (5.6)	3 (2.3)	2 (1.8)	
**Have you ever gone to a fortune-teller?**					12.508, *p* = 0.006*
Yes	43 (23.5)	13 (10.5)	22 (16.5)	12 (10.6)	
No	136 (74.3)	105 (84.7)	106 (79.7)	100 (88.5)	
Missing	4 (2.2)	6 (4.8)	5 (3.8)	1 (0.9)	
**Do you believe that some days are lucky while others are not?**					4.416, *p* = 0.220
Yes	105 (57.4)	55 (44.4)	75 (56.4)	58 (51.3)	
No	73 (39.9)	59 (47.6)	52 (39.1)	53 (46.9)	
Missing	5 (2.7)	10 (8.1)	6 (4.5)	2 (1.8)	
**Do you believe that signs may predict upcoming misfortune?**					6.194, *p* = 0.103
Yes	80 (43.7)	43 (34.7)	57 (42.9)	37 (32.7)	
No	93 (50.8)	74 (59.7)	71 (53.4)	74 (65.5)	
Missing	10 (5.5)	7 (5.6)	5 (3.8)	2 (1.8)	

HCs, healthy controls; MS, multiple sclerosis; PD, Parkinson’s disease; ^a^results of Monte Carlo test are presented; *p < 0.05; **p < 0.001.

**Table 3:** Frequency of belief in omens and superstitions explored in the study. A detailed list of omens and superstitions is provided in the Supplementary Appendix.

**No.**		**Epilepsy (*n* = 183)**	**PD (*n* = 124)**	**MS (*n* = 133)**	**HC (*n* = 113)**	**Test and *p* value**
	**Meaning ascribed to omens (*n*, %)**					
O1^a^	Positive	48 (26.2)	15 (12.1)	17 (12.8)	35 (31)	20.609, *p* = 0.001*
	Negative	4 (2.2)	3 (2.4)	2 (1.5)	0 (0)	
	None	130 (71)	92 (74.2)	109 (82)	77 (68.1)	
	Missing	1 (0.5)	14 (11.3)	5 (3.8)	1 (0.9)	
O2	Positive	42 (23)	19 (15.3)	22 (16.5)	23 (20.4)	10.874, *p* = 0.092
	Negative	31 (16.9)	12 (9.7)	11 (8.3)	11 (9.7)	
	None	109 (59.6)	81 (65.3)	98 (73.7)	78 (69)	
	Missing	1 (0.5)	12 (9.7)	2 (1.5)	1 (0.9)	
O3^a^	Positive	29 (15.8)	8 (6.5)	15 (11.3)	13 (11.5)	20.196, *p* = 0.002*
	Negative	16 (8.7)	1 (0.8)	5 (3.8)	1 (0.9)	
	None	136 (74.3)	100 (80.6)	109 (82)	98 (86.7)	
	Missing	2 (1.1)	15 (12.1)	4 (3)	1 (0.9)	
O4^a^	Positive	56 (30.6)	13 (10.5)	20 (15)	13 (11.5)	29.648, *p* < 0.001**
	Negative	6 (3.3)	2 (1.6)	3 (2.3)	0 (0)	
	None	117 (63.9)	95 (76.6)	105 (78.9)	99 (87.6)	
	Missing	4 (2.2)	14 (11.3)	5 (3.8)	1 (0.9)	
O5^a^	Positive	5 (2.7)	4 (3.2)	2 (1.5)	1 (0.9)	12.312, *p* = 0.045*
	Negative	79 (43.2)	33 (26.6)	37 (27.8)	40 (35.4)	
	None	97 (53)	77 (62.1)	90 (67.7)	71 (62.8)	
	Missing	2 (1.1)	10 (8.1)	4 (3)	1 (0.9)	
O6^a^	Positive	2 (1.1)	0 (0)	1 (0.8)	0 (0)	14.444, *p* = 0.009*
	Negative	77 (42.1)	26 (21)	44 (33.1)	40 (35.4)	
	None	99 (54.1)	85 (68.5)	86 (64.7)	73 (64.6)	
	Missing	5 (2.7)	13 (10.5)	2 (1.5)	0 (0)	
O7^a^	Positive	4 (2.2)	1 (0.8)	1 (0.8)	0 (0)	19.755, *p* = 0.002*
	Negative	79 (43.2)	23 (18.5)	46 (34.6)	39 (34.5)	
	None	97 (53)	86 (69.4)	84 (63.2)	74 (65.5)	
	Missing	3 (1.6)	14 (11.3)	2 (1.5)	0 (0)	
O8^a^	Positive	8 (4.4)	4 (3.2)	1 (0.8)	2 (1.8)	6.351, *p* = 0.380
	Negative	66 (36.1)	35 (28.2)	42 (31.6)	34 (30.1)	
	None	107 (58.5)	74 (59.7)	87 (65.4)	77 (68.1)	
	Missing	2 (1.1)	11 (8.9)	3 (2.3)	0 (0)	
O9^a^	Positive	6 (3.3)	1 (0.8)	2 (1.5)	1 (0.9)	5.275, *p* = 0.513
	Negative	64 (35)	35 (28.2)	47 (35.3)	35 (31)	
	None	105 (57.4)	80 (64.5)	81 (60.9)	77 (68.1)	
	Missing	8 (4.4)	8 (6.5)	3 (2.3)	0 (0)	
	**Belief in superstitious statements (*n*, %)**					
S1	Yes	56 (30.6)	12 (9.7)	33 (24.8)	26 (23)	16.344, *p* = 0.001*
	No	123 (67.2)	100 (80.6)	95 (71.4)	86 (76.1)	
	Missing	4 (2.2)	12 (9.7)	5 (3.8)	1 (0.9)	
S2	Yes	52 (28.4)	33 (26.6)	36 (27.1)	29 (25.7)	0.419, *p* = 0.936
	No	127 (69.4)	83 (66.9)	94 (70.7)	84 (74.3)	
	Missing	4 (2.2)	8 (6.5)	3 (2.3)	0 (0)	
S3	Yes	68 (37.2)	34 (27.4)	49 (36.8)	35 (31)	2.875, *p* = 0.411
	No	114 (62.3)	80 (64.5)	82 (61.7)	78 (69)	
	Missing	1 (0.5)	10 (8.1)	2 (1.5)	0 (0)	
S4	Yes	53 (29)	15 (12.1)	25 (18.8)	19 (16.8)	13.046, *p* = 0.005*
	No	128 (69.9)	98 (79)	105 (78.9)	94 (83.2)	
	Missing	2 (1.1)	11 (8.9)	3 (2.3)	0 (0)	
S5	Yes	67 (36.6)	19 (15.3)	34 (25.6)	26 (23)	16.043, *p* = 0.001*
	No	114 (62.3)	94 (75.8)	96 (72.2)	87 (77)	
	Missing	2 (1.1)	11 (8.9)	3 (2.3)	0 (0)	
S6	Yes	26 (14.2)	7 (5.6)	15 (11.3)	8 (7.1)	7.065, *p* = 0.070
	No	153 (83.6)	108 (87.1)	115 (86.5)	105 (92.9)	
	Missing	4 (2.2)	9 (7.3)	3 (2.3)	0 (0)	
S7	Yes	13 (7.1)	1 (0.8)	5 (3.8)	9 (8)	7.894, *p* = 0.048*
	No	167 (91.3)	111 (89.5)	125 (94)	104 (92)	
	Missing	3 (1.6)	12 (9.7)	3 (2.3)	0 (0)	
S8	Yes	48 (26.2)	13 (10.5)	31 (23.3)	34 (30.1)	13.554, *p* = 0.004*
	No	131 (71.6)	102 (82.3)	100 (75.2)	78 (69)	
	Missing	4 (2.2)	9 (7.3)	2 (1.5)	1 (0.9)	
S9	Yes	26 (14.2)	4 (3.2)	11 (8.3)	18 (15.9)	12.447, *p* = 0.006*
	No	154 (84.2)	109 (87.9)	120 (90.2)	94 (83.2)	
	Missing	3 (1.6)	11 (8.9)	2 (1.5)	1 (0.9)	
S10	Yes	28 (15.3)	6 (4.8)	17 (12.8)	28 (24.8)	17.546, *p* = 0.001*
	No	151 (82.5)	106 (85.5)	113 (85)	84 (74.3)	
	Missing	4 (2.2)	12 (9.7)	3 (2.3)	1 (0.9)	
S11	Yes	49 (26.8)	13 (10.5)	31 (23.3)	22 (19.5)	11.073, *p* = 0.011*
	No	133 (72.7)	102 (82.3)	98 (73.7)	91 (80.5)	
	Missing	1 (0.5)	9 (7.3)	4 (3)	0 (0)	
S12	Yes	23 (12.6)	1 (0.8)	12 (9)	7 (6.2)	13.763, *p* = 0.003*
	No	158 (86.3)	110 (88.7)	117 (88)	106 (93.8)	
	Missing	2 (1.1)	13 (10.5)	4 (3)	0 (0)	
S13	Yes	73 (39.9)	26 (21)	37 (27.8)	39 (34.5)	11.364, *p* = 0.010*
	No	107 (58.5)	88 (71)	93 (69.9)	74 (65.5)	
	Missing	3 (1.6)	10 (8.1)	3 (2.3)	0 (0)	
S14	Yes	56 (30.6)	15 (12.1)	35 (26.3)	20 (17.7)	16.105, *p* = 0.001*
	No	124 (67.8)	101 (81.5)	94 (70.7)	93 (82.3)	
	Missing	3 (1.6)	8 (6.5)	4 (3)	0 (0)	
S15	Yes	73 (39.9)	25 (20.2)	47 (35.3)	30 (26.5)	13.975, *p* = 0.003*
	No	105 (57.4)	89 (71.8)	84 (63.2)	82 (72.6)	
	Missing	5 (2.7)	10 (8.1)	2 (1.5)	1 (0.9)	
S16	Yes	60 (32.8)	23 (18.5)	51 (38.3)	26 (23)	13.131, *p* = 0.004*
	No	120 (65.6)	88 (71)	80 (60.2)	87 (77)	
	Missing	3 (1.6)	13 (10.5)	2 (1.5)	0 (0)	
S17	Yes	61 (33.3)	18 (14.5)	39 (29.3)	44 (38.9)	16.264, *p* = 0.001
	No	118 (64.5)	95 (76.6)	91 (68.4)	69 (61.1)	
	Missing	4 (2.2)	11 (8.9)	3 (2.3)	0 (0)	
S18	Yes	61 (33.3)	10 (8.1)	32 (24.1)	37 (32.7)	26.030, *p* < 0.001*
	No	118 (64.5)	103 (83.1)	98 (73.7)	76 (67.3)	
	Missing	4 (2.2)	11 (8.9)	3 (2.3)	0 (0)	

HCs, healthy controls; MS, multiple sclerosis; O, omen; PD, Parkinson’s disease; S, superstition; ^a^results of Monte Carlo test are presented, *p < 0.05, **p < 0.001.

On average, patients with PD (*n* = 105, Md = 0, IQR = 0–3) and MS (*n* = 124, Md = 1, IQR = 0–5) less often ascribed meaning to omens than PWE (*n* = 171, Md = 3, IQR = 0–6), H (3)=21.543, *p* < 0.001. Individuals with PD (*n* = 103, Md = 0, IQR = 0–4) also believed in less superstitions than HCs (*n* = 109, Md = 2, IQR = 1–10), PWE (*n* = 163, Md = 3, IQR = 0–8) or patients with MS (*n* = 124, Md = 2, IQR = 0–8), H (3)=23.032, p < 0.001. Complete SI scores were collected for 479 (86.6%) participants. The calculated SI was lower among patients with PD (*n* = 96, Md = 1, IQR = 0–5.75) than among those with epilepsy (*n* = 155, Md = 6, IQR = 1–14), MS (*n* = 120, Md = 4, IQR = 0–12) or HCs (*n* = 108, Md = 4.5, IQR = 1–10), H (3)=26.780, *p* < 0.001.

The SI was higher among women (Md = 5.5, IQR = 0–13 vs. Md = 2, IQR = 0–8, Z = -3.862, p < 0.001) and participants living in villages (H (2)=7.062, *p* = 0.029), but did not depend on the place of birth (H (2)=1.766, *p* = 0.414), relationship status (H (4)=4.079, *p* = 0.395), employment (H (3)=4.154, *p* = 0.245) or source of income (H (3)=6.479, *p* = 0.090). The SI was inversely correlated with age (*ρ* = −0.156, *p* = 0.001), the level of education (*ρ* = −0.189, *p* < 0.001) and average monthly income (*ρ* = −0.170, *p* < 0.001), but its relationship with the number of children (ρ = −0.062, *p* = 0.180) was not statistically significant. In the negative binomial regression model (*n* = 394, likelihood ratio *χ*^2^ = 35.178, *p* < 0.001), only sex had a statistically significant association with the SI (Table 4). If age was removed from the model, both female sex (*β* = 0.422, OR = 1.525, 95% CI = 1.148 to 2.026) and Parkinson’s disease (*β* = −0.428, OR = 0.652, 95% CI = 0.432 to 0.984) were significant predictors of the SI. In a regression model created for the group of participants with PD alone (*n* = 85, likelihood ratio *χ*^2^ = 2.149, *p* = 0.828), none of the previous demographic variables were statistically significant (*p* > 0.05).

**Table 4:** Results of a negative binomial regression model with the superstition index as the dependent variable.

**Independent variable**	***β***	**Standard error**	**Wald *χ*** ^**2**^	***P***	**Odds ratio**	**95% Confidence Interval**
**Intercept**	1.827	0.3565	26.255	<0.001**	6.212	3.089 to 12.494
**Sex**						
Female	0.423	0.1450	8.491	0.004*	1.526	1.148 to 2.028
Male	ref.					
**Age**	<0.001	0.0052	0.004	0.950	1.000	0.990 to 1.011
**Place of residence (higher = more rural)**	0.126	0.1028	1.510	0.219	1.135	0.928 to 1.388
**Average monthly income**	−0.058	0.0747	0.599	0.439	0.944	0.815 to 1.093
**Level of education**	−0.073	0.0489	2.249	0.134	0.929	0.844 to 1.023
**Group**						
Epilepsy	0.195	0.1826	1.146	0.284	1.216	0.850 to 1.739
PD	−0.438	0.2610	2.813	0.093	0.645	0.387 to 1.077
MS	0.080	0.2117	0.144	0.704	1.084	0.716 to 1.641
HCs	ref.					
**Negative binomial**	1.606	0.1470				

HCs, healthy controls; MS, multiple sclerosis; PD, Parkinson’s disease.

Two-step cluster analysis that included study participants from all groups (cluster quality = 0.5 (fair), Supplementary Figure S1) resulted in two clusters comprised of “non-believers” (*n* = 253 (52.8%), the majority of the cluster members disagree that most of the listed omens or superstitions have any meaning) and “believers” (*n* = 226 (47.2%), the majority of the cluster agrees with many of the statements).

Subgroups of the study sample (PWE, patients with PD, MS or HCs) were categorized in either two or three clusters.

Among PWE (cluster quality = 0.4 (fair), Supplementary Figure S2), 92 (59.4%) were grouped as “non-believers” and 63 (40.6%) as “believers (>50% of the cluster believed in 18 (66.7%) statements).”

Patients with PD (cluster quality>0.5 (good), Supplementary Figure S3) were classified in three clusters. One of them was a “non-believer” cluster (28, 29.2%), another – a “believer” cluster (18, 18.8%, >50% of members believed in 12 (44.4%) statements). The largest cluster among participants with PD may be perceived as an “extreme non-believer” group (50, 52.1%), in which 98–100% of members do not believe in any of the omens or superstitions.

Clusters in MS [cluster quality>0.5 (good), Supplementary Figure S4] were like those of PWE: 63 (52.5%) in the “non-believer” cluster and 57 (47.5%) in the “believer” cluster (>50% of members believed in 13 (48.2%) statements). A similar cluster structure [cluster quality = 0.4 (fair), Supplementary Figure S5] was also present for HCs [56 (51.9%) in the “non-believer” cluster and 52 (48.1%) in the “believer” cluster].

## 4 Discussion

In the presented survey we aimed to test whether patients with PD are less superstitious (i.e., less likely to agree with statements with misattribution of properties of one ontological category to another) than PWE, patients with MS and HCs (H_1_), and whether PWE are more superstitious than the other groups (H_2_). Results provided evidence for H_1_ but not H_2_: patients with PD had overall lower scores of the SI and were more often categorized in “extreme non-believer” and “non-believer” rather than “believer” groups in cluster analysis. The tendency for patients with PD to be less superstitious was also supported by results of the negative binomial regression (used because the SI was treated as a count variable) after adjustment for sex, the level of education, place of residence, and socioeconomic status.

To the best of our knowledge, there are currently no published studies assessing superstitious thinking in PD or providing underlying mechanisms (or possible confounders) to explain our results. If superstitious behavior in humans is supposed to largely depend on conditional learning, it may be hypothesized to result from chaotic dopamine release within the mesolimbic system (23). According to this hypothesis, superstition is regarded to be the result of adventitious conditioning, as observed by Skinner in pigeons who developed idiosyncratic behavior in response to a scheduled presentation of food (24). Therefore, at least in theory, deficient dopamine release in PD could prevent the formation of superstitious tendencies. Excessive dopaminergic transmission (e.g., in case of the impulse control disorder), on the other hand, could promote superstitious behavior, similarly as in problem gamblers (25, 26). Chronic use of ketamine, a competitive antagonist of NMDA receptors with complex pharmacodynamics, as well as polydrug use have been shown to promote superstitious conditioning in humans, supporting the potential plasticity of this process and its dependence on long-term changes in neurotransmission (27, 28). However, it is important to note that none of the biological or pharmacological explanations were directly explored in our study. It is also difficult to see how the dopamine-associated theory would apply for non-personal (cultural) superstitions which were predominant in our questionnaire. Moreover, it remains unclear whether the hypothesized underlying mechanisms are the same for conditioned superstitious *behavior* and for superstitious *belief* that was tested in the current study (29, 30). Finally, superstitious conditioning is also eased in case of hippocampal lesions, but no association between superstitious belief and epilepsy was found in our study (29, 31, 32). This points toward the possibility that various other factors, such as severity of the underlying disease, psychological, societal and family aspects, rather than neurophysiological changes could significantly contribute to our findings. One of the most obvious confounders in our study is age which is naturally higher in individuals with PD and may therefore reflect a vastly different cultural and societal setting present at the time when such individuals were younger and formed their belief systems, including superstitiousness. Therefore, our results should be replicated in a setting where the superstitiousness of patients with PD is compared to the one of healthy individuals of the same age, religion and cultural background.

Current results indicated no major difference in religiosity among the study groups. While we used only three questions to evaluate religiosity and did not perform and in-depth analysis of this multidimensional part of human life, our preliminary findings further suggest that religiosity should be treated separately from superstitious beliefs (18). While most studies find individuals with PD to be less religious than controls, some suggest that PD has no clear influence on religiosity (6, 7, 33–40). In semantic priming studies, patients with PD were shown to have worse activation of religious concepts (e.g., prime – “sacred,” target – “sense spirit,” “become holy”) and thus dysfunction in right-sided striatal-prefrontal networks has been hypothesized (6, 34, 36–38). A recent report of neuroimaging data proposes that lesions in individuals with parkinsonism overlap with those associated with decreased spirituality (41). However, data from standardized questionnaires remains contrasting with some studies showing loss of spirituality but not faith in PD (39, 40). Investigations of religiosity and/or spirituality in both PD and epilepsy vary greatly in their methodology and are often argued to be insufficiently rigorous in their approach of confounders (34, 42). Interestingly, positive answers to items related to astrology (belief in horoscopes) and magical thinking (visiting a fortune-teller) but not religion were more frequent in epilepsy than PD, MS or HCs, further supporting the vision that superstition, religiosity and spirituality are probably different psychological constructs.

The results of the current study should be interpreted cautiously because of its different limitations. First, the comparison of subjects with PD, epilepsy or MS is subject to various confounding factors, such as disability, disease duration or medication use, which were unaccounted for in the current study. The three disorders have vastly different etiologies, pathogenetic pathways as well as dissimilar social, cognitive and mental health ramifications. While we attempted to adjust our analysis for demographic and socioeconomic variables, other disease-specific aspects or comorbidities that make the groups heterogeneous may also significantly influence our findings. Importantly, part of the group of healthy controls was composed of healthy patients’ relatives, making this group prone to similarities in cultural, religious and social aspects with other respondents instead of the general population. Moreover, items selected for inclusion in the list of omens and superstitions was created *ad hoc* to better explore beliefs that are thought to be widespread locally – given the lack of up-to-date academic articles reporting on superstitious beliefs in the country, there was no gold standard against which our selection could be externally validated or thoroughly adapted to the population. Therefore, our list of superstitions and omens was based on news articles and websites and the selected phenomena were evaluated by the author group only for face validity, dimensionality and construct validity. However, the broader external validity of our questionnaire remains unknown, thus reducing the reproducibility and generalizability of the study. According to the data of the 2021 Census, 74.2% of residents in Lithuania consider themselves to be Roman Catholics, 6.1% are non-believers, 3.8% are Orthodox while other religious communities comprise a minority of the population (43). Because of the methodological and cultural differences, our findings may also not be directly comparable to previously reported data in this field of research.

## 5 Conclusion

We reported results of a survey focused on superstitious beliefs in patients with three different chronic neurological disorders – epilepsy, MS and PD. After accounting for sex, education, and socioeconomic status, our data indicates that individuals with Parkinson’s disease tend to have lower belief in superstitions compared to other patient groups. The association between PD and decreased superstitiousness should be explored further, especially by considering PD-specific disease factors.

## Data availability statement

The original contributions presented in the study are included in the article/Supplementary material, further inquiries can be directed to the corresponding author/s.

## Ethics statement

Ethical review and approval was not required for the study on human participants in accordance with the local legislation and institutional requirements. Written informed consent from the patients/participants or patients/participants' legal guardian/next of kin was not required to participate in this study in accordance with the national legislation and the institutional requirements.

## Author contributions

RM: Conceptualization, Data curation, Formal analysis, Investigation, Methodology, Project administration, Supervision, Visualization, Writing – original draft, Writing – review & editing. RKi: Investigation, Visualization, Writing – review & editing. RKa: Investigation, Visualization, Writing – review & editing. KP: Conceptualization, Data curation, Formal analysis, Methodology, Visualization, Writing – original draft, Writing – review & editing.

## References

[1] Feigin VL Nichols E Alam T Bannick MS Beghi E Blake N. Global, regional, and national burden of neurological disorders, 1990–2016: a systematic analysis for the global burden of disease study 2016. Lancet Neurol. (2019) 18:459–80. doi: 10.1016/S1474-4422(18)30499-X, PMID: 30879893 PMC6459001

[2] Deuschl G Beghi E Fazekas F Varga T Christoforidi KA Sipido E. The burden of neurological diseases in Europe: an analysis for the global burden of disease study 2017. Lancet Public Health. (2020) 5:e551–67. doi: 10.1016/S2468-2667(20)30190-0 33007212

[3] Feigin VL Vos T Nichols E Owolabi MO Carroll WM Dichgans M. The global burden of neurological disorders: translating evidence into policy. Lancet Neurol. (2020) 19:255–65. doi: 10.1016/S1474-4422(19)30411-9, PMID: 31813850 PMC9945815

[4] Olesen J Gustavsson A Svensson M Wittchen HU Jönsson B. The economic cost of brain disorders in Europe. Eur J Neurol. (2012) 19:155–62. doi: 10.1111/j.1468-1331.2011.03590.x 22175760

[5] Hesdorffer DC. Comorbidity between neurological illness and psychiatric disorders. CNS Spectr. (2016) 21:230–8. doi: 10.1017/S1092852915000929 26898322

[6] Butler PM McNamara P. Comment on: Parkinson’s disease, religion, and spirituality. Mov Disord Clin Pract. (2016) 3:518. doi: 10.1002/mdc3.12330, PMID: 30363544 PMC6178695

[7] Redfern C Mason SL Barker RA Coles A. Parkinson’s disease and spirituality. Neuro Rehabilitation. (2020) 46:31–9. doi: 10.3233/NRE-192947 32039874

[8] Rigon IB Calado GA Linhares LS Cantu PLM Moritz JLW Wolf P. Religiosity and spirituality in patients with epilepsy. Arq Neuropsiquiatr. (2019) 77:335–40. doi: 10.1590/0004-282x20190055, PMID: 31188997

[9] Tedrus GMAS Fonseca LC Fagundes TM da Silva GL. Religiosity aspects in patients with epilepsy. Epilepsy Behav. (2015) 50:67–70. doi: 10.1016/j.yebeh.2015.06.003, PMID: 26133113

[10] Korczyn AD Schachter SC Amlerova J Bialer M van Emde Boas W Brázdil M. Third international congress on epilepsy, brain and mind: part 1. Epilepsy Behav. (2015) 50:116–37. doi: 10.1016/j.yebeh.2015.06.044, PMID: 26276417 PMC5256665

[11] McCrae N Whitley R. Exaltation in temporal lobe epilepsy: neuropsychiatric symptom or portal to the divine? J Med Humanit. (2014) 35:241–55. doi: 10.1007/s10912-014-9294-4, PMID: 25017116

[12] Lindeman M Dvedholm AM. What’s in a term? Paranormal, superstitious, magical and supernatural beliefs by any other name would mean the same. Rev Gen Psychol. (2012) 16:241–55. doi: 10.1037/a0027158.supp

[13] Risen JL. Believing what we do not believe: acquiescence to superstitious beliefs and other powerful intuitions. Psychol Rev. (2016) 123:182–207. doi: 10.1037/rev0000017 26479707

[14] Kahneman D Frederick S. A model of heuristic judgment, *the Cambridge handbook of thinking and reasoning*. NewYork, NY, USA: Cambridge University Press (2005). 267–293

[15] Kahneman D Frederick S. Representativeness revisited: Attribute substitution in intuitive judgment, *heuristics and biases*. NewYork, NY, USA: Cambridge University Press (2012). 49–81.

[16] Gallup. Thirteen percent of Americans bothered to stay on hotels’ 13th floor, Available at: https://news.gallup.com/poll/26887/Thirteen-Percent-Americans-Bothered-Stay-Hotels-13th-Floor.aspx. (2007)

[17] Gallup. One in four Americans superstitious, Available at: https://news.gallup.com/poll/2440/One-Four-Americans-Superstitious.aspx. (2000)

[18] Martin del Campo Rios J. Religion and superstition through a cognitive perspective: examining the relationship of religious and superstitious beliefs to cognitive processes. University of Leicester. Thesis. (2015). Available at: https://hdl.handle.net/2381/32224

[19] Daprati E Sirigu A Desmurget M Nico D. Superstitious beliefs and the associative mind. Conscious Cogn. (2019) 75:102822. doi: 10.1016/j.concog.2019.102822, PMID: 31557563

[20] Brugger P Mohr C. The paranormal mind: how the study of anomalous experiences and beliefs may inform cognitive neuroscience. Cortex. (2008) 44:1291–8. doi: 10.1016/j.cortex.2008.05.008 18632092

[21] Payne EH Gebregziabher M Hardin JW Ramakrishnan V Egede LE. An empirical approach to determine a threshold for assessing overdispersion in Poisson and negative binomial models for count data. Commun Stat Simul Comput. (2018) 47:1722–38. doi: 10.1080/03610918.2017.1323223, PMID: 30555205 PMC6290908

[22] Kaufman L Rousseeuw PJ. Finding groups in data: an introduction to cluster analysis. New Jersey: John Wiley & Sons (1990). 88 p.

[23] Shaner A. Delusions, superstitious conditioning and chaotic dopamine neuro-dynamics. Med Hypotheses. (1999) 52:119–23. doi: 10.1054/mehy.1997.0656, PMID: 10340292

[24] Skinner BF. “Superstition” in the pigeon. J Exp Psychol. (1948) 38:168–72. doi: 10.1037/h0055873, PMID: 18913665

[25] Joukhador J Blaszczynski A Maccallum F. Superstitious beliefs in gambling among problem and non-problem gamblers: preliminary data. J Gambl Stud. (2004) 20:171–80. doi: 10.1023/B:JOGS.0000022308.27774.2b 15060332

[26] Vilas D Pont-Sunyer C Tolosa E. Impulse control disorders in Parkinson’s disease. Parkinsonism Relat Disord. (2012) 18:S80–4. doi: 10.1016/s1353-8020(11)70026-8 22166463

[27] Mion G Villevieille T. Ketamine pharmacology: an update (pharmacodynamics and molecular aspects, recent findings). CNS Neurosci Ther. (2013) 19:370–80. doi: 10.1111/cns.12099, PMID: 23575437 PMC6493357

[28] Freeman TP Morgan CJA Klaassen E Das RK Stefanovic A Brandner B. Superstitious conditioning as a model of delusion formation following chronic but not acute ketamine in humans. Psychopharmacology. (2009) 206:563–73. doi: 10.1007/s00213-009-1564-x, PMID: 19436994

[29] Brugger P Viaud-Delmon I. Superstitiousness in obsessive-compulsive disorder. (2010). 250–254. Available at: www.dialogues-cns.org 10.31887/DCNS.2010.12.2/pbrugger PMC3181957 20623929

[30] Brugger P Graves RE. Testing vs. believing hypotheses: magical ideation in the judgement of contingencies. Cogn Neuropsychiatry. (1997) 2:251–72. doi: 10.1080/135468097396270, PMID: 25419793

[31] Devenport LD Holloway FA. The Rat’s resistance to superstition: role of the hippocampus. J. Comp. Physiol. Psychol. (1980). 94:691–705. doi: 10.1037/h0077703 7190980

[32] Brugger P Dowdy MA Graves RE. From superstitious behavior to delusional thinking: the role of the hippocampus in misattributions of causality. Med Hypotheses. (1994). 43:397–402. doi: 10.1016/0306-9877(94)90015-9 7739412

[33] Otaiku AI. Religiosity and risk of Parkinson’s disease in England and the USA. J Relig Health. (2022) 62:4192–208. doi: 10.1007/s10943-022-01603-8, PMID: 35763200 PMC10682218

[34] Redfern C Coles A. Parkinson’s disease, religion, and spirituality. Mov Disord Clin Pract. (2015) 2:341–6. doi: 10.1002/mdc3.12206 30363549 PMC6178734

[35] McNamara P Raymon Durso R Brown A. Religiosity in patients with Parkinson’s disease. Neuropsychiatr Dis Treat. (2006) 2:241–348. 19412480 10.2147/nedt.2006.2.3.341 PMC2671825

[36] Butler PM McNamara P Durso R. Deficits in the automatic activation of religious concepts in patients with Parkinsons disease. J Int Neuropsychol Soc. (2010) 16:252–61. doi: 10.1017/S1355617709991202, PMID: 19958570

[37] Butler PM McNamara P Durso R. Side of onset in Parkinson’s disease and alterations in religiosity: novel behavioral phenotypes. Behav Neurol. (2011) 24:133–41. doi: 10.3233/BEN-2011-0282, PMID: 21606574 PMC5377949

[38] Butler PM McNamara P Ghofrani J Durso R. Disease-associated differences in religious cognition in patients with Parkinson’s disease. J Clin Exp Neuropsychol. (2011) 33:917–28. doi: 10.1080/13803395.2011.575768, PMID: 21656414

[39] Giaquinto S Bruti L Dall'Armi V Palma E Spiridigliozzi C. Religious and spiritual beliefs in outpatients suffering from Parkinson disease. Int J Geriatr Psychiatry. (2011) 26:916–22. doi: 10.1002/gps.2624, PMID: 21845593

[40] Kéri S Kelemen O. Faith unchanged: spirituality, but not christian beliefs and attitudes, is altered in newly diagnosed parkinson’s disease. Religions. (2016) 7:73. doi: 10.3390/rel7060073

[41] Ferguson MA Schaper FLWVJ Cohen A Siddiqi S Merrill SM Nielsen JA. A neural circuit for spirituality and religiosity derived from patients with brain lesions. Biol Psychiatry. (2022) 91:380–8. doi: 10.1016/j.biopsych.2021.06.016, PMID: 34454698 PMC8714871

[42] Bone I Dein S. Religion, spirituality, and epilepsy. Epilepsy Behav. (2021) 122:108219. doi: 10.1016/j.yebeh.2021.108219, PMID: 34343961

[43] Statistics Lithuania. Results of the 2021 population and housing census of the Republic of Lithuania. Nationality, native language and religion. Available at: https://osp.stat.gov.lt/2021-gyventoju-ir-bustu-surasymo-rezultatai/tautybe-gimtoji-kalba-ir-tikyba (Accessed February 10, 2024).

