# Supplementary material for: Belief in omens and superstitions among patients with chronic neurological disorders
Source: Front Public Health. 2024 Mar 7;12:1331254. doi: 10.3389/fpubh.2024.1331254 (PMC10958788; doi:10.3389/fpubh.2024.1331254)

Supplementary Material

#
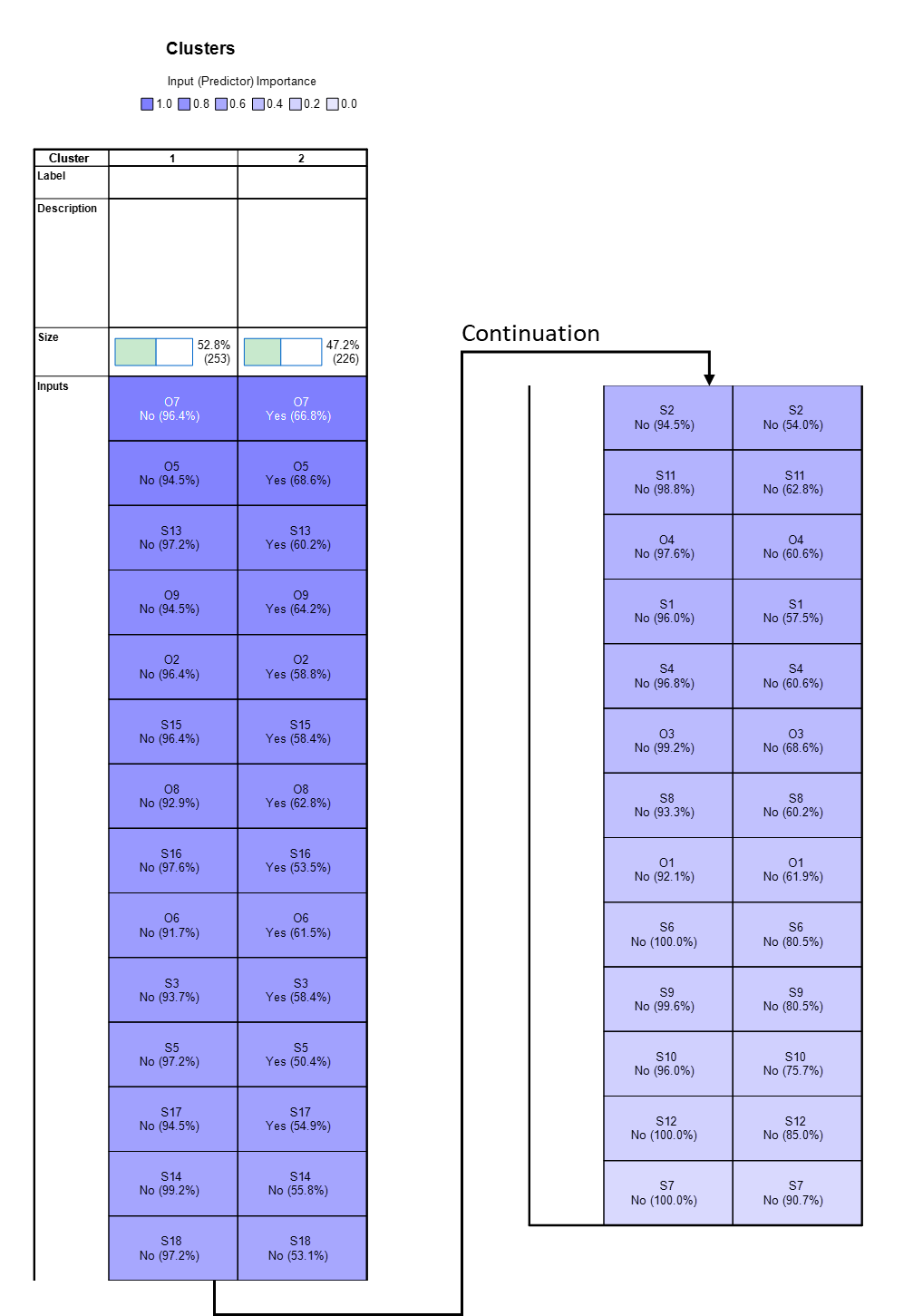
Supplementary Figure S1. Two-step cluster analysis (all participants). S – superstitious statement, O – omen


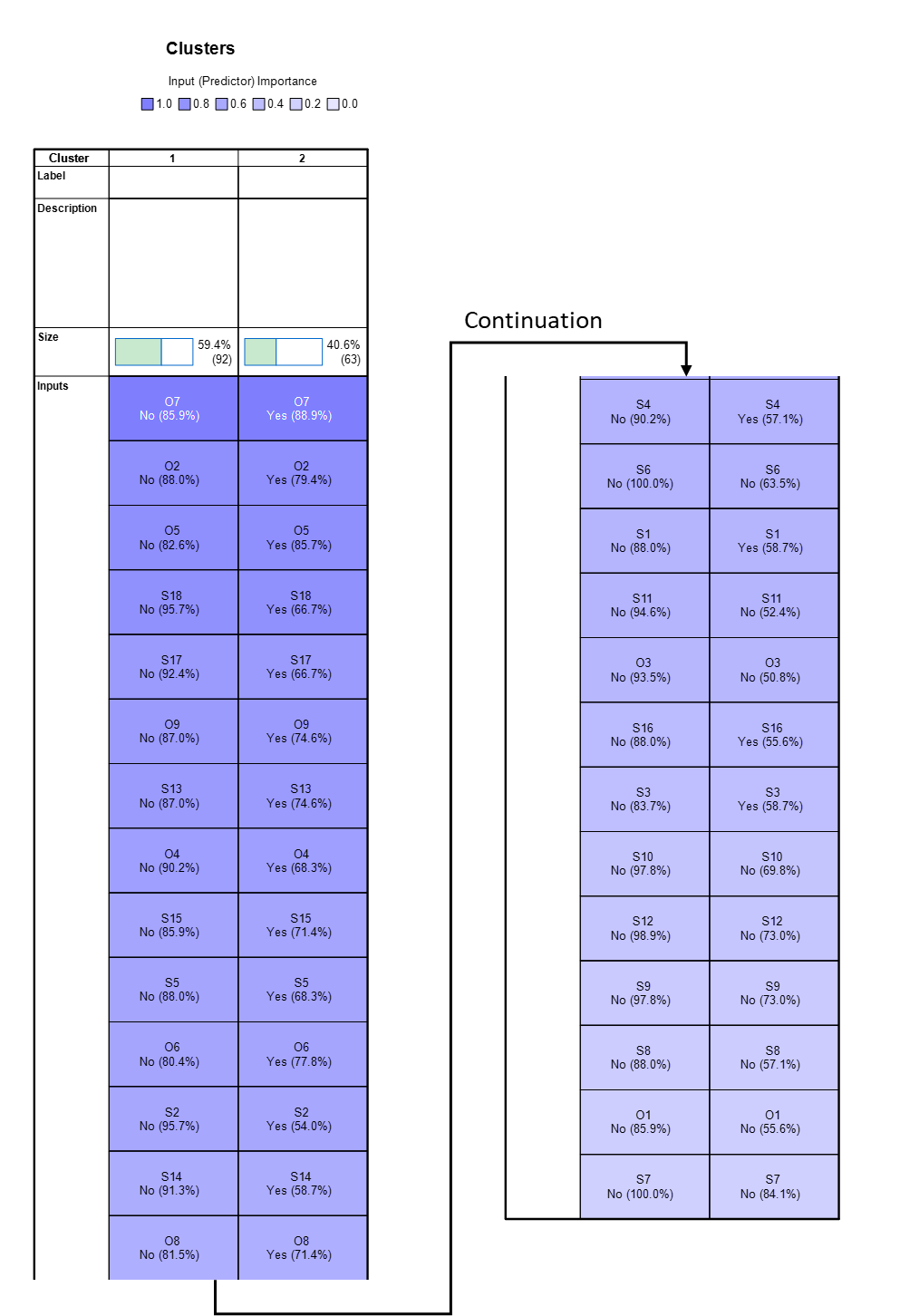
**Supplementary Figure S2.** Two-step cluster analysis (patients with epilepsy). S – superstitious statement, O – omen.

**Supplementary Figure S3.** Two-step cluster analysis (Parkinson’s disease). S – superstitious statement, O – omen.


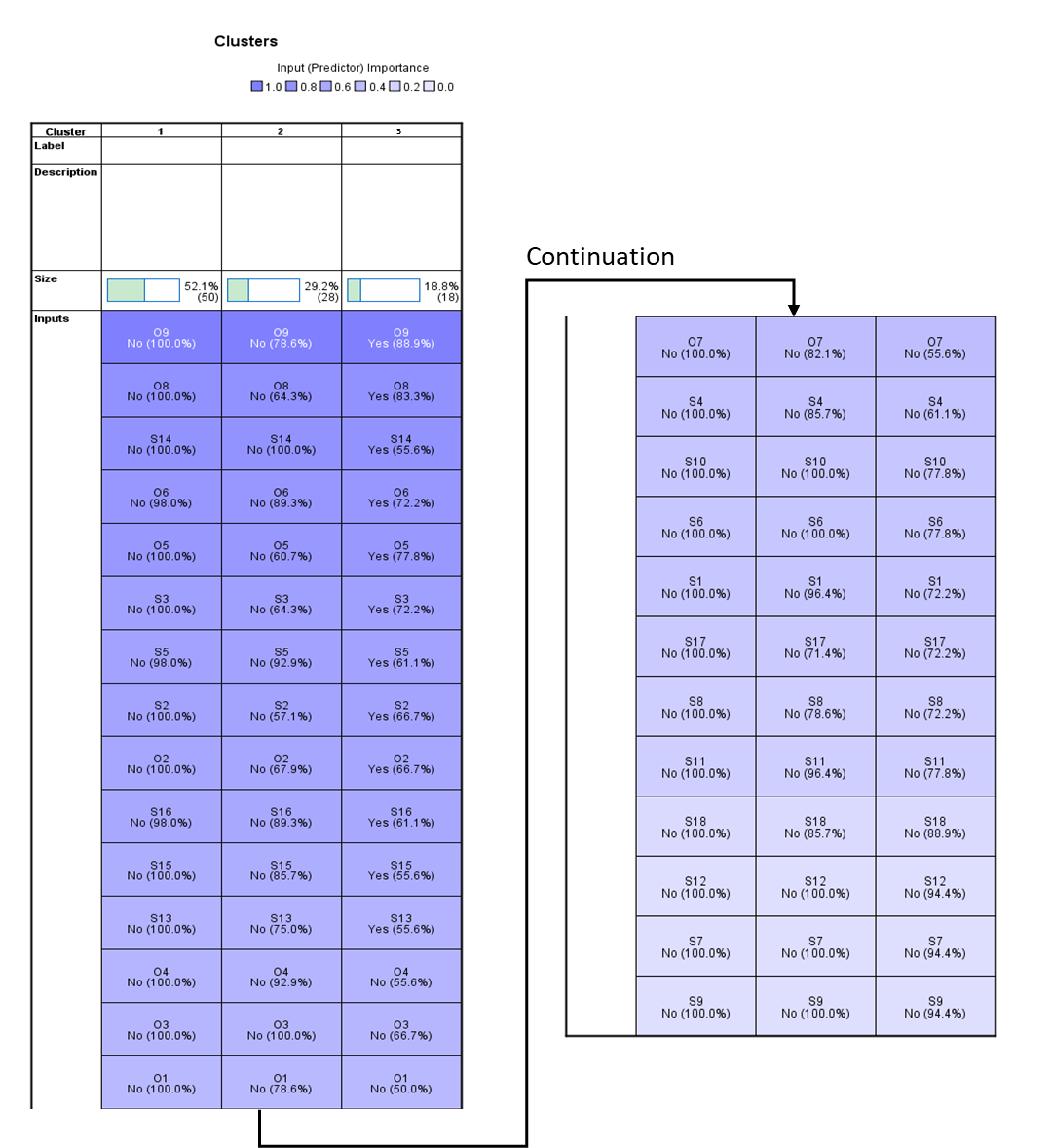


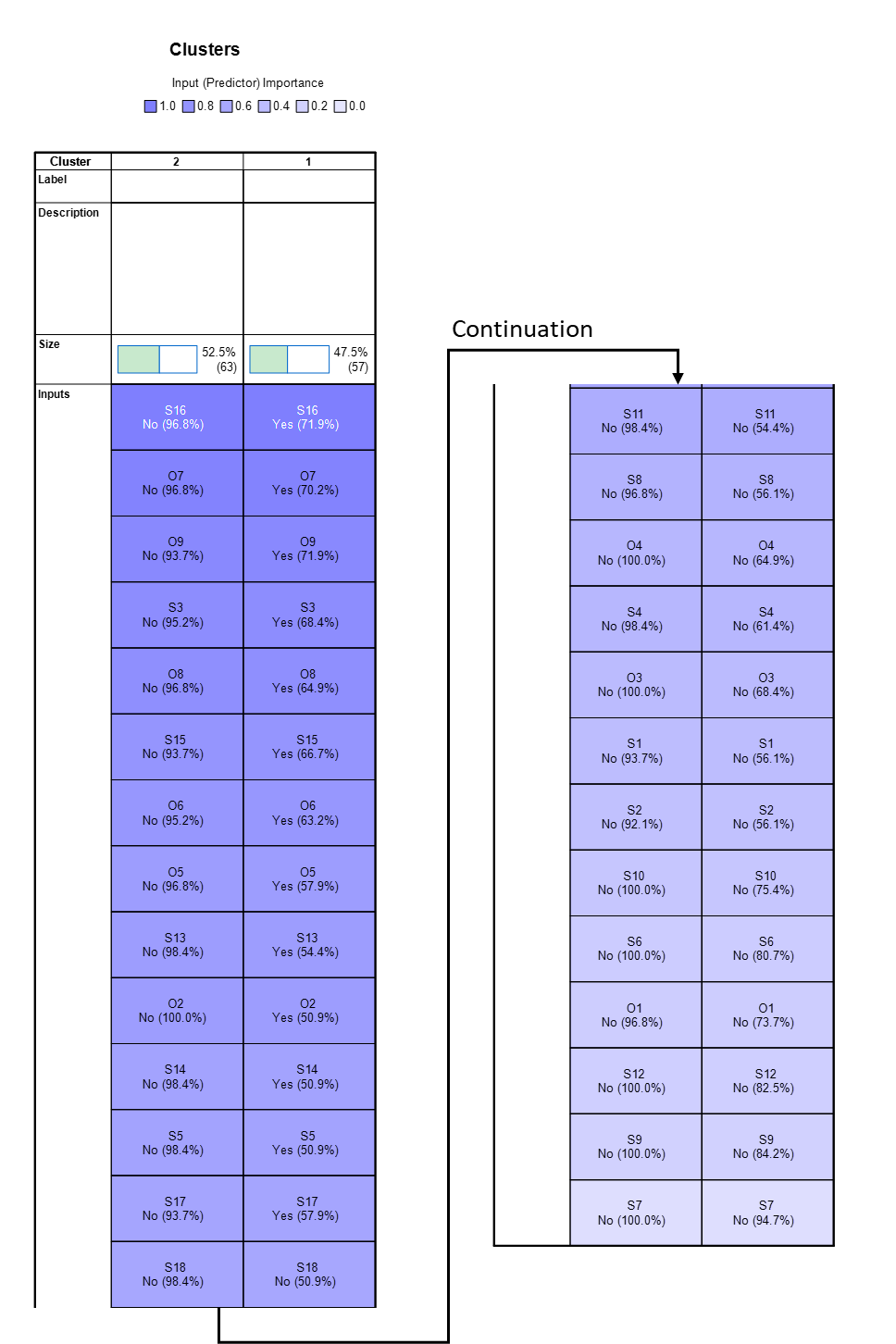
**Supplementary Figure S4.** Two-step cluster analysis (patients with multiple sclerosis). S – superstitious statement, O – omen.

**Supplementary Figure S5**. Two-step cluster analysis (healthy controls). S – superstitious statement, O – omen.


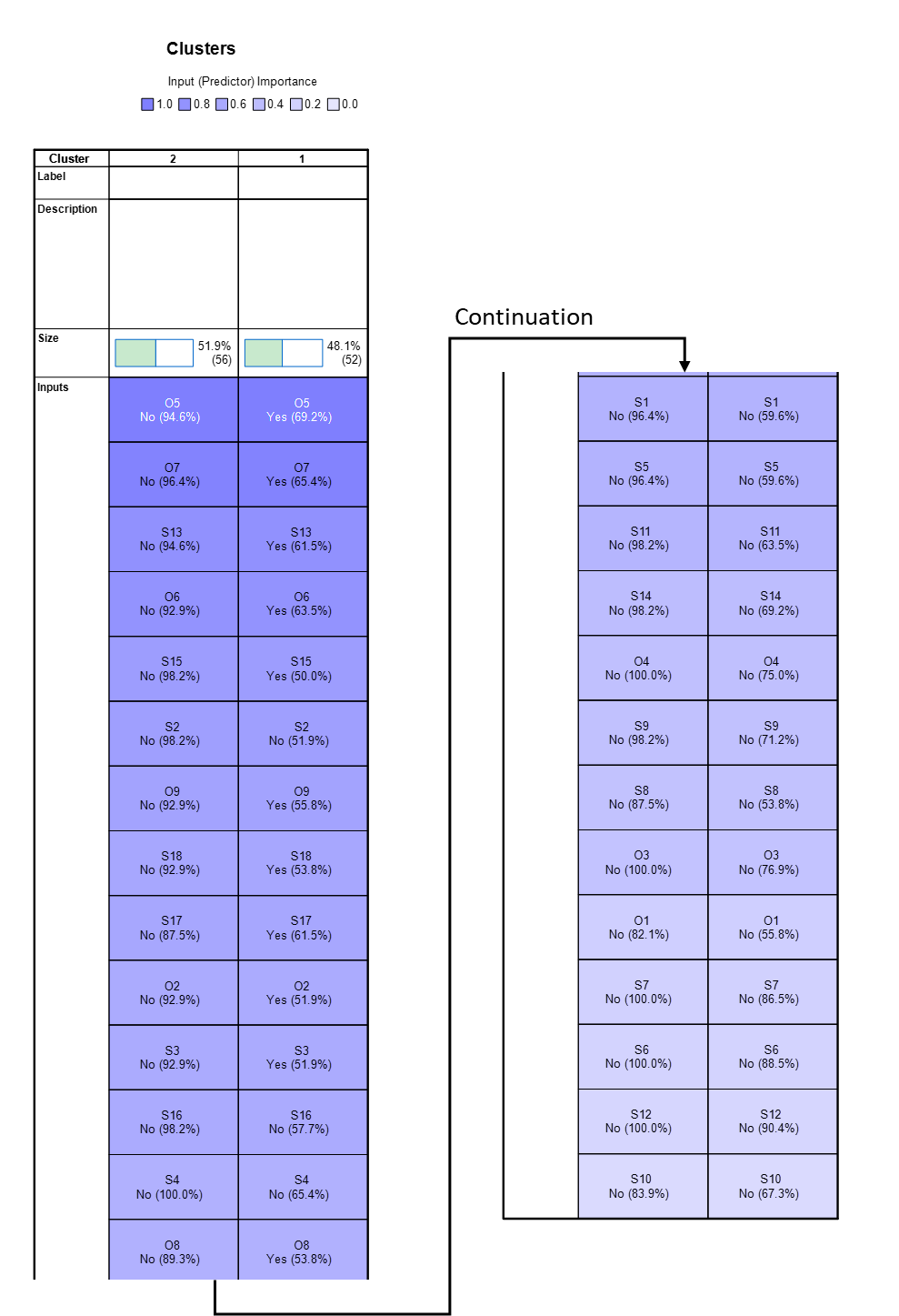

Supplement: Supplementary file 1 [file Data_Sheet_1.docx]
