# Supplementary material for: Belief in omens and superstitions among patients with chronic neurological disorders
Source: Front Public Health. 2024 Mar 7;12:1331254. doi: 10.3389/fpubh.2024.1331254 (PMC10958788; doi:10.3389/fpubh.2024.1331254)
Supplement: Supplementary file 2 [file Data_Sheet_2.docx]

Supplementary Material

**Supplementary Appendix.** Omens and superstitions included in the questionnaire form.

| **Item number** | **Please mark (X) what the following signs mean to you:** | **…is a good sign** | **…is a bad sign** | **…has no meaning** |
| --- | --- | --- | --- | --- |
| O1 | Finding a coin on the sidewalk… |  |  |  |
| O2 | Breaking of a glass bowl… |  |  |  |
| O3 | Seeing a crawling spider… |  |  |  |
| O4 | A ladybug landing on you… |  |  |  |
| O5 | A black cat crossing the street… |  |  |  |
| O6 | Losing a wedding ring… |  |  |  |
| O7 | Breaking a mirror… |  |  |  |
| O8 | A bird hitting your window… |  |  |  |
| O9 | Dreaming of falling teeth… |  |  |  |
|  | **Please mark (X) if you believe in the following statements:** | | **Yes, I do** | **No, I do not** |
| S1 | If you wish upon a shooting star, your wish will be granted | |  |  |
| S2 | To have a coin in one's pocket when the cuckoo's call is first heard in spring ensures wealth for the rest of the year | |  |  |
| S3 | One must look in the mirror at home after coming to back to pick something that was forgotten | |  |  |
| S4 | One can expect misfortune on Friday the 13^th^ | |  |  |
| S5 | If your ears burn, someone is talking about you | |  |  |
| S6 | Spilling salt will result in quarrels at home | |  |  |
| S7 | If you pick up a comb, you will experience shame in the future | |  |  |
| S8 | Your wishes will not be granted after telling them to someone | |  |  |
| S9 | You may not marry for seven years if you sit at the corner of a table | |  |  |
| S10 | You should not thank if someone wishes good luck | |  |  |
| S11 | You should not put your bag (or purse) on the floor – you will lose wealth | |  |  |
| S12 | Washing dishes as a guest brings bad luck | |  |  |
| S13 | You should not shake hands over a doorway – this brings conflict | |  |  |
| S14 | You should not thank if someone gives you medication – it will not work | |  |  |
| S15 | You should not gift an empty wallet, at least some cash must be inside | |  |  |
| S16 | You should not gift sharp objects – you should “sell” them at least for a small amount of cash | |  |  |
| S17 | Knocking on wood wards off bad luck | |  |  |
| S18 | Spitting three times over the left shoulder wards off bad luck | |  |  |
